# Use of Tetrabenazine in Huntington Disease Patients on Antidepressants or with Advanced Disease: Results from the TETRA-HD Study

**DOI:** 10.1371/currents.RRN1283

**Published:** 2011-11-13

**Authors:** Ray Dorsey, Kevin Biglan, Shirley Eberly, Peggy Auinger, Alicia Brocht, Chizoba C. Umeh, David Oakes, Kathleen Clarence-Smith, Frederick Marshall, Ira Shoulson, Samuel Frank

**Affiliations:** ^*^Neurologist, Johns Hopkins University; ^†^Department of Neurology, University of Rochester, Rochester, NY, USA; ^‡^University of Rochester Medical Center, Rochester NY; ^#^Movement Disorders Fellow, Johns Hopkins University; ^††^Partner, KM Pharmaceutical Consulting; ^‡‡^Associate Professor of Neurology, University of Rochester School of Medicine and Dentistry; ^§§^Professor of Neurology, Pharmacology & Human Science. Director, Program for Regulatory Science & Medicine (PRSM) and ^¶¶^Doctor

## Abstract

The safety and effectiveness of tetrabenazine in different sub-populations of Huntington disease (HD) is not known. In this study, we evaluated the safety of tetrabenazine in individuals on an antidepressant and its effectiveness in advanced HD. Tetrabenazine was not associated with an increased incidence of depressed mood among those taking antidepressants and was effective at reducing chorea in those with advanced HD.

**Background**: The safety and effectiveness of tetrabenazine in different sub-populations of Huntington disease (HD) is not known.  We evaluated its safety in individuals on an antidepressant and its effectiveness in advanced HD.    

**Methods: **The TETRA-HD study was a randomized, placebo-controlled, double-blind study of 84 individuals with HD who were randomized 2:1 to receive tetrabenazine (54 participants) or placebo (30 participants).  We used data from the 12-week randomized controlled study of tetrabenazine to evaluate the incidence of depressed mood either as an adverse event or as a two points or greater worsening on the “depressed mood” item of the Unified Huntington Disease Rating Scale.  We also evaluated the effectiveness of tetrabenazine in reducing chorea in advanced HD.  Advanced HD was defined as a baseline maximum total chorea score of 19 or greater or as a total functional capacity score of seven or less. Only individuals who were randomized to tetrabenazine (n=54) were included in these analyses.     

**Results:**At baseline, 27 (56%) of the 48 participants randomized to tetrabenazine who completed the trial were taking an antidepressant.  The incidence of depressed mood did not differ between those taking (15%) and not taking (5%) an antidepressant (p=0.37), and the proportion of individuals experiencing a substantial worsening in their mood scores also did not differ (7% v. 10%; p=1.00).  

            Based on chorea, 14 of the 54 (26%) tetrabenazine participants enrolled had advanced HD, and based on function, 22 (41%) had advanced HD. Chorea declined by 8.1 units among those with baseline scores 19 or greater compared to 4.3 units for those with scores under 19 (p<0.01), but the relative rates of decline did not differ (p=0.62).  The decline in chorea did not differ by HD severity as measured by function (p=0.20).  

**Conclusion: **In this *post hoc *analysis, tetrabenazine was not associated with an increased incidence of depressed mood among those taking antidepressants and was effective at reducing chorea in those with advanced HD.  Larger, prospective studies are needed to confirm these findings.

## Introduction

      Tetrabenazine is an efficacious treatment for chorea associated with Huntington disease (HD). In a large randomized controlled study of tetrabenazine in HD (TETRA-HD), individuals randomized to tetrabenazine had a 5.0 point reduction in their chorea severity compared with a 1.5 point reduction in those receiving placebo[1]. However, despite its efficacy, questions remain about its safety and benefit in different sub-populations, including those who are taking an antidepressant and those with advanced HD.

      Tetrabenazine works by reversibly inhibiting the human vesicular monoamine transporter 2 leading to the depletion of monoamines from nerve terminals[2]. Among the monoamines depleted is dopamine, which is implicated as the primary neurotransmitter involved in the etiology of chorea. Within the central nervous system, tetrabenazine causes preferential depletion of dopamine from nerve terminals. 

        In addition to dopamine, tetrabenazine also depletes serotonin albeit at likely higher doses. In rats, following subcutaneous administration of tetrabenazine, the effective dose for striatal dopamine depletion is approximately 0.4 mg/kg subcutaneous, whereas the effective dose for norepinephrine and serotonin depletion is approximately 2 mg/kg subcutaneous. Similar preferential dopamine depletion was observed in post-mortem brains from Huntington disease patients with or without a history of tetrabenazine treatment[3] [4].  Due to this effect, the use of tetrabenazine in individuals with depression or on anti-depressants (which achieve their effect via an intact monoamine system)[5], is a potential concern.  In one study on the long-term effects of tetrabenazine in hyperkinetic movement disorders, depression was noted as a side effect in 15% of patients[6].  In addition, some antidepressants (e.g., paroxetine) are CYP2D6 inhibitors, which are responsible for the metabolism of the active metabolites of tetrabenazine[7].   Thus these drugs could raise the serum levels of the active metabolites of tetrabenazine and may alter its safety and efficacy profile.

      Advanced HD is another important sub-population for anti-chorea treatment.  While chorea is an early motor sign of HD and has functional and social consequences, its physical consequences in the later stages of HD can be large[8]. As disease burden increases so do motor signs[9], yet the efficacy of tetrabenazine in this population has not been well-established.  Because of questions about the safety of tetrabenazine use in individuals taking an anti-depressant and its efficacy in advanced HD, we investigated these questions in supplemental analyses of the TETRA-HD study.

## Methods

*TETRA-HD Study*

      The TETRA-HD study was a randomized, placebo-controlled, double-blind study of 84 individuals with HD.  Individuals were randomized 2:1 to receive tetrabenazine (54 participants) or placebo (30 participants) and followed for 12 weeks.  The dose of tetrabenazine was increased over seven weeks to a maximum of 100 mg/day, until the desired anti-choreic effect was achieved or intolerable adverse events occurred.  Research participants had a total functional capacity (TFC) of at least six and a total maximal chorea of at least ten as measured by the Unified Huntington’s Disease Rating Scale (UHDRS). Individuals with “disabling depression” were excluded but individuals on stable doses of antidepressants were permitted to enroll in the study[1]. Only participants (n=54) randomized to tetrabenazine were included in these analyses.

*Assessing depressed mood in individuals taking an antidepressant*

      For the primary safety analysis, we evaluated the incident development of depressed mood either as an adverse event coded as “depressed mood” or as a two point or greater worsening on the “depressed mood” item of the UHDRS (frequency multiplied by severity) among participants who were randomized to tetrabenazine and who completed the 12-week study (n=48).   We assessed the development of depressed mood in those taking an antidepressant at baseline compared to those who were not.  We repeated this analysis in the entire intention-to-treat cohort randomized to tetrabenazine (n=54).  

*Assessing efficacy in individuals with advanced HD*

      We used two definitions for advanced HD.  The first was individuals with a baseline total chorea score of 19 or greater.  The second was individuals with a TFC of seven or lower.  Changes in chorea scores were compared for those with advanced HD to those without advanced HD.  For one of the primary efficacy analyses, all participants randomized to tetrabenazine (n=54) were included, and for missing values, we carried forward the last observation.  The analyses were then repeated for participants who completed the study.

      In the other primary efficacy analysis, we compared clinical global improvement scores and new adverse events between the two groups. Because the outcomes depended upon the amounts of time that subjects were observed, these analyses were limited to those subjects who completed the study (n=48).  However, they were also repeated using the entire cohort.

*Statistical analysis*

      Comparisons of depressed mood in participants on and not on antidepressants at baseline were made using Fisher’s exact tests.  Changes in chorea scores were evaluated in participants whose disease was more advanced or less advanced at baseline using multiple regression models that included baseline TFC or baseline chorea scores.  To evaluate the relative decline in chorea score, the data were log-transformed.  Cochran-Armitage trend tests were used to compare global improvement scores and numbers of adverse events for subjects whose disease status was more advanced and less advanced at baseline.

## Results

*Incidence of depressed mood in individuals taking an antidepressant and tetrabenazine*

      At baseline, 27 (56%) of the 48 participants randomized to tetrabenazine who completed the trial were taking an antidepressant.  The incidence of depressed mood as an adverse event did not differ between those taking (15%) and not taking (5%) an antidepressant (p=0.37), and the proportion of individuals experiencing a substantial worsening in their mood scores did not differ (7% v. 10%; p=1.00).  The results were similar when examining all 54 participants randomized to tetrabenazine (**Table 1**).  

**Table 1.**  Depression in participants on and not on antidepressants at baseline  

**Table:** 

**Study population**		**N**	**Incident adverse event of depressed mood **	Increase in depressed mood by at least 2 points*
**Those who completed the study**		**48**	** **	** **
	On antidepressants	27	4 (15%)	2 (7%)
	Not on antidepressants	21	1 (5%)	2 (10%)
	P-value**		0.37	1.00
**All participants**		**54**	** **	** **
	On antidepressants	30	5 (17%)	3 (10%)
	Not on antidepressants	24	1 (4%)	3 (13%)
	P-value**		0.21	1.00

 *   As measured by the Unified Huntington's Disease Rating Scale

**  P-values were obtained using Fisher’s exact tests

  *Effect of tetrabenazine on individuals with advanced HD*

      At baseline, based on chorea scores, 14 of the 54 (26%) participants enrolled had advanced HD, and based on function, 22 (41%) had advanced HD. The decline in chorea was 8.1 units for those with a baseline chorea of 19 or greater compared to 4.3 units for those with a baseline chorea of between 10 and 18 (p<0.01). However, the relative rates of decline in chorea between the two groups did not differ (p=0.62).  Results for participants who completed the study were similar.

      Among the 48 participants completing the study, the 12-week clinical global improvement scores between the two groups did not differ (p=0.95).  The proportion of individuals with at least one adverse event among those with advanced HD was 75% compared to 80% for those without advanced HD.  The numbers of adverse events reported did not differ between the two groups (p=0.18).  Results were similar when the entire tetrabenazine cohort was included in the analyses.

      Defining advanced HD by total functional capacity scores generated similar results (**Table 2**).****

**Table 2.**  Efficacy and safety of tetrabenazine by baseline disease severity  

**Table:** 

**Study population**		**N**	Mean (SD) change in chorea score*	Median global improvement**	Experienced at least one adverse event**
**Those who completed the study**		**48**			
	Baseline chorea score of 19 or more	12	-7.6 (6.0)	2.5	9 (75%)
	Baseline chorea score of less than 19	36	-4.5 (3.8)	3.0	29 (80%)
	P-value		0.065	0.95	0.18
**All participants**		**54**			
	Baseline chorea score of 19 or more	14	-8.1 (5.7)	2.5	9 (64%)
	Baseline chorea of less than 19	40	-4.3 (3.8)	3.0	31 (78%)
	P-value		0.009	0.91	0.09
**Those who completed the study**		**48**			
	Total Functional Capacity of 7 or less	20	-6.7 (5.0)	2.0	16 (80%)
	Total Functional Capacity of more than 7	28	-4.3 (4.1)	3.0	22 (79%)
	P-value		0.20	0.64	0.44
**All participants**		**54**			
	Total Functional Capacity of 7 or less	22	-6.6 (5.0)	2.0	16 (73%)
	Total Functional Capacity of more than 7	32	-4.4 (4.2)	3.0	24 (75%)
	P-value		0.20	0.86	0.33

SD = standard deviation  * P-values were obtained from adjusted multiple regression models.  ** P-values were obtained using Cochran-Armitage trend tests.

## **Discussion**

      Based on supplemental analyses of the TETRA-HD study, the incidence of depressed mood with tetrabenazine does not differ when used in individuals who are taking an antidepressant compared to those who are not taking one. While not necessarily generalizable to a larger population, these results show that in our study population, tetrabenazine can be used with caution in those individuals who are taking an antidepressant. This observation can be used as the basis for future studies with larger numbers of research participants evaluating the safety of tetrabenazine in individuals with HD and depression. The safety of tetrabenazine in individuals with HD and who are on an antidepressant is important as approximately 45% of individuals with HD take an antidepressant[9].  Additionally, we conclude that tetrabenazine is efficacious in reducing chorea in those with advanced HD where chorea can be quite disabling.

      These results have several limitations.  First, they are based on a *post hoc *analysis of the data from the study.  Second, even though the TETRA-HD is the largest randomized controlled study of tetrabenazine, the sample sizes in these analyses were small (subsets of approximately 50) leading to limited statistical power and the potential for a type II error (i.e. false negative findings).  Third, individuals in clinical trials generally receive more frequent and detailed assessments, which may not be the case in clinical practice. Fourth, the duration of observation in the TETRA-HD study was short, only 12 weeks, and provides limited opportunity to identify potential adverse consequences.  In clinical practice, the occurrence of depression over 29 months is reported as 15%. In an open-label study of tetrabenazine up to 80 weeks, depressed mood was reported as the second most common adverse event[10].  Fifth, participants in the TETRA-HD study had to be “independently ambulatory” and had to have a total functional capacity greater than five[1].  As such, the efficacy of tetrabenazine in very advanced HD, especially in those requiring institutional care, remains to be assessed.

      Because of these limitations, the safety and efficacy of tetrabenazine in practice in different sub-populations require future study.  One approach is to use large prospective longitudinal studies to determine both the safety and efficacy of tetrabenazine and other treatments.  While such studies lack the detailed assessments of a clinical trial, their size gives greater power to detect differences and potentially less common events, such as suicide and suicide attempts, and more closely reflect (but do not mirror) clinical practice.  Fortunately, in HD, data from such studies exist.  Both the REGISTRY study led by the European Huntington’s Disease Network[9] and the Cooperative Huntington’s Observational Research Trial led by the Huntington Study Group[11] contain data on over one thousand individuals with HD, each with observation periods extending to five years or more.  These studies can better inform the safety and efficacy of tetrabenazine, provide additional guidance to clinicians, give information about the use of other concomitant medications used to suppress chorea, and even inform proper labeling of the drug by regulators.  These studies and other prospective investigations can better determine the safety and efficacy of tetrabenazine and other treatments for HD in different sub-populations.  Until such assessments are conducted, these *post hoc *analyses demonstrate that within our study population, tetrabenazine can be used with caution in individuals taking an anti-depressant at baseline and in individuals with more advanced HD. Larger scale assessments in practice will be important to determine whether these findings hold true.

## Acknowledgements

We thank Paige Nichols, BA for her assistance in preparing the manuscript.

## Funding information

Lundbeck provided funding for this study.

## Competing interests

Drs. Dorsey, Biglan, Marshall, Clarence-Smith, Shoulson, and Frank have all served as independent consultants to Lundbeck.

## Corresponding author

Ray Dorsey

600 N. Wolfe St., Meyer 6-181D

Baltimore, MD 21287

ray.dorsey@jhmi.edu

410.614.5991 (Tel)

410.502.6737 (Fax)

The TETRA-HD study was approved by the Research Subjects Review Board at the University of Rochester and by the Institutional Review Board at each participating site. Written informed consent was obtained from all study participants.
